# Effects of Partial Cow–Calf Contact and Social Companions on Health, Performance, and Behavior of Calves

**DOI:** 10.3390/ani16162571

**Published:** 2026-08-18

**Authors:** Z. Panahi, E. Ghasemi, A. H. Mahdavi, M. Sadeghi, F. Ahmadi

**Affiliations:** 1 Department of Animal Sciences, College of Agriculture, Isfahan University of Technology, Isfahan 84156-83111, Iranghasemi@cc.iut.ac.ir (E.G.); mahdavi@iut.ac.ir (A.H.M.); 2 Department of Eco-Friendly Livestock Science, Institute of Green Bio Science and Technology, Seoul National University, Pyeongchang 25354, Republic of Korea

**Keywords:** health, performance, contact, separation, calf

## Abstract

Provision of cow–calf contact (CCC) in combination with pair housing may enhance welfare and stimulate greater intake of both milk and starter feed, thereby improving the performance and health of calves reared under cold conditions. This study was conducted to investigate the effects of combining partial daily CCC (5 h/d) with pair housing (19 h/d) in dairy calves reared in outdoor pens during winter, when the mean ambient temperature was 6.2 ± 0.4 °C throughout the study period. A total of 30 Holstein calves (birth weight = 37.4 ± 3.4 kg) were enrolled at birth. Fourteen calves received daily dam contact and were pair-housed (DC-PH), while the remaining 16 calves had no dam contact (NC); of these, 8 were pair-housed (NC-PH) and 8 were individually housed (NC-IH). Dam contact was maintained until day 60, after which all calves were weaned and group-housed until day 80. Milk consumption was greater in DC than NC calves (7.4 vs. 5.5 L/d). During the pre-weaning period, body weight and average daily gain did not differ between treatments. However, DC-PH calves had greater body weight at day 80 and higher average daily gain between days 60 and 80 than NC-PH calves (*p* = 0.01 and *p* = 0.02, respectively). Pre-weaning starter feed intake and feed efficiency did not differ across treatment groups. Dry matter digestibility was significantly greater in NC-PH than in NC-IH calves (87.7% vs. 81.4%; *p* = 0.03), but no significant effects were observed on NDF and DM digestibility between NC-PH and DC-PH calves. On day 54 (the pre-weaning phase), compared with NC-PH calves, DC-PH calves had higher blood glucose (*p* = 0.05), lower cortisol (*p* = 0.02), tended to have lower βHBA concentrations (*p* = 0.06), and a greater ruminal protozoal count on day 35 (*p* = 0.01). Total health score tended to be higher in DC-PH than NC-PH calves (*p* = 0.06) and was higher in NC-IH than in NC-PH calves (*p* = 0.05). During an 8-h behavioral recording prior to weaning, DC-PH calves were observed less frequently performing non-nutritive oral behaviors, such as licking the stall or fences and sucking on other calves, and tended to be more frequently standing, contacting other calves, playing alone, walking, and ruminating than NC-PH calves. After weaning, DC-PH calves were observed more frequently eating, resting, walking, and engaging in social interactions than NC-PH calves. Likewise, after weaning, NC-IH calves were observed more frequently playing, vocalizing, and walking, but less eating than NC-PH calves. Overall, our findings indicated that while social interaction (NC-IH vs. NC-PH) significantly improved dry matter digestibility and health, the addition of dam contact (DC-PH) maintained nutrient digestibility at levels comparable to NC-PH, but tended to reduce health status. Also, calves in the DC-PH treatment, which voluntarily consumed more milk than NC calves, exhibited greater post-weaning growth, more favorable behavioral responses, and improved pre-weaning metabolic profiles (lower cortisol and βHBA concentrations and higher blood glucose). These findings indicate that the partial dam contact management system, together with greater voluntary milk intake, was associated with enhanced post-weaning performance and metabolic status, as well as differences in rumen microbial development. However, as milk intake was not standardized across treatments, these responses cannot be attributed solely to maternal contact. Overall, under the conditions of this study, partial dam contact represents a feasible management strategy to support calf welfare and development, provided that appropriate environmental management is implemented to minimize disease risks.

## Simple Summary

Early separation of dairy calves from their mothers is a common industry practice, but it has raised concerns regarding animal welfare. Providing calves with limited daily contact with their mothers, alongside pair housing, may improve their well-being by satisfying natural social and nursing needs. This study evaluated the effects of partial (5 h/d) cow–calf contact (CCC) combined with pair housing during winter. Our findings indicate that calves raised with CCC demonstrated lower physiological stress levels before weaning and achieved better growth after weaning compared to those raised without dam contact. While these calves also exhibited more natural social behaviors, they experienced higher health challenges during the cold winter period. Our results suggest that partial CCC combined with pair housing may be a promising compromise to improve calf welfare and performance, although careful health management is needed in winter.

## 1. Introduction

Although the neonatal and infancy periods represent a relatively brief phase of a calf’s life cycle, they are critical for establishing the foundation for long-term physical, behavioral, and cognitive development [1]. In commercial dairy production systems, calves are frequently exposed to diverse social, managerial, and environmental stressors that can collectively compromise their growth and long-term health. Currently, individual housing remains the most prevalent management practice, often implemented to facilitate calf monitoring and mitigate undesirable behaviors such as cross-sucking; however, this calf management practice is labor-intensive and has been linked to social isolation, potentially impairing cognitive and behavioral maturation [1,2]. Furthermore, the maternal deprivation and peer isolation associated with individual housing can negatively affect calf welfare [3,4], a concern that has gained increasing public attention as routine cow–calf separation at birth faces growing societal scrutiny and raises expectations for more welfare-oriented management practices [5,6].

In contrast, rearing calves in groups during the milk-feeding period can promote natural social interactions and stimulate essential behaviors [7]. Prior studies suggest that group-housed calves may achieve higher weaning weights due to increased feed intake, a phenomenon likely driven by social learning and peer facilitation during feeding [7,8,9]. Despite these recognized benefits, many dairy operations continue to prioritize individual housing, transitioning to group housing only post-weaning [10]. Although continuous maternal contact has been shown to enhance social skills and resilience, it presents practical challenges, such as reduced saleable milk volume and potential delays in rumen development due to excessive milk intake [11,12]. To mitigate these drawbacks, partial dam-contact systems, particularly half-day contact, have been proposed as a means to facilitate gradual weaning, promote solid feed intake, and improve rumen development [11,13,14].

The efficacy of these management systems is further influenced by environmental conditions, particularly thermal stress. Rearing young calves in outdoor facilities during winter months significantly increases energy requirements; insufficient nutrient supply under such conditions can compromise growth, immunity, and overall health [15,16]. Due to their high surface area-to-body mass ratio and limited subcutaneous fat, newborn calves are particularly susceptible to cold stress when ambient temperatures fall below their lower critical temperature, which has been estimated at approximately 15 °C for calves under 3 wk of age [17] and 5 °C for older calves [18]. Under such challenging thermal conditions, social and maternal factors may serve as crucial mitigants. For example, huddling behavior in group-housed calves can aid in heat conservation [16], while maternal presence may stimulate activity and early feeding, indirectly supporting thermoregulation [19].

Although the individual advantages of partial dam contact and social housing are documented, their synergistic impact, particularly when integrated to mitigate the physiological challenges of the cold season, remains poorly understood. We hypothesized that calves provided with partial daily dam contact combined with pair housing during the milk-feeding period would contribute to superior growth performance, rumen development, and positive behavioral outcomes compared to conventionally reared calves. Therefore, the objective of the present study was to evaluate the effects of partial daily dam contact and pair housing on the health, growth performance, rumen development, and behavior of Holstein calves reared during the cold season.

## 2. Materials and Methods

The study was conducted at the dairy research facility of Isfahan University of Technology (Lavark, Isfahan, Iran) from October 2022 to February 2023. The experiment was designed to be conducted during the cold season, given that low environmental temperatures represent a high-risk scenario for calf health, thermoregulation, and growth performance. However, seasonal effects were not directly evaluated in the present study. All animal procedures were reviewed by the Animal Care and Use Committee of Isfahan University of Technology prior to the commencement of the study. All procedures complied with the guidelines of the Iranian Council of Animal Care [20].

### 2.1. Animals and Treatment Groups

**Figure 1:**
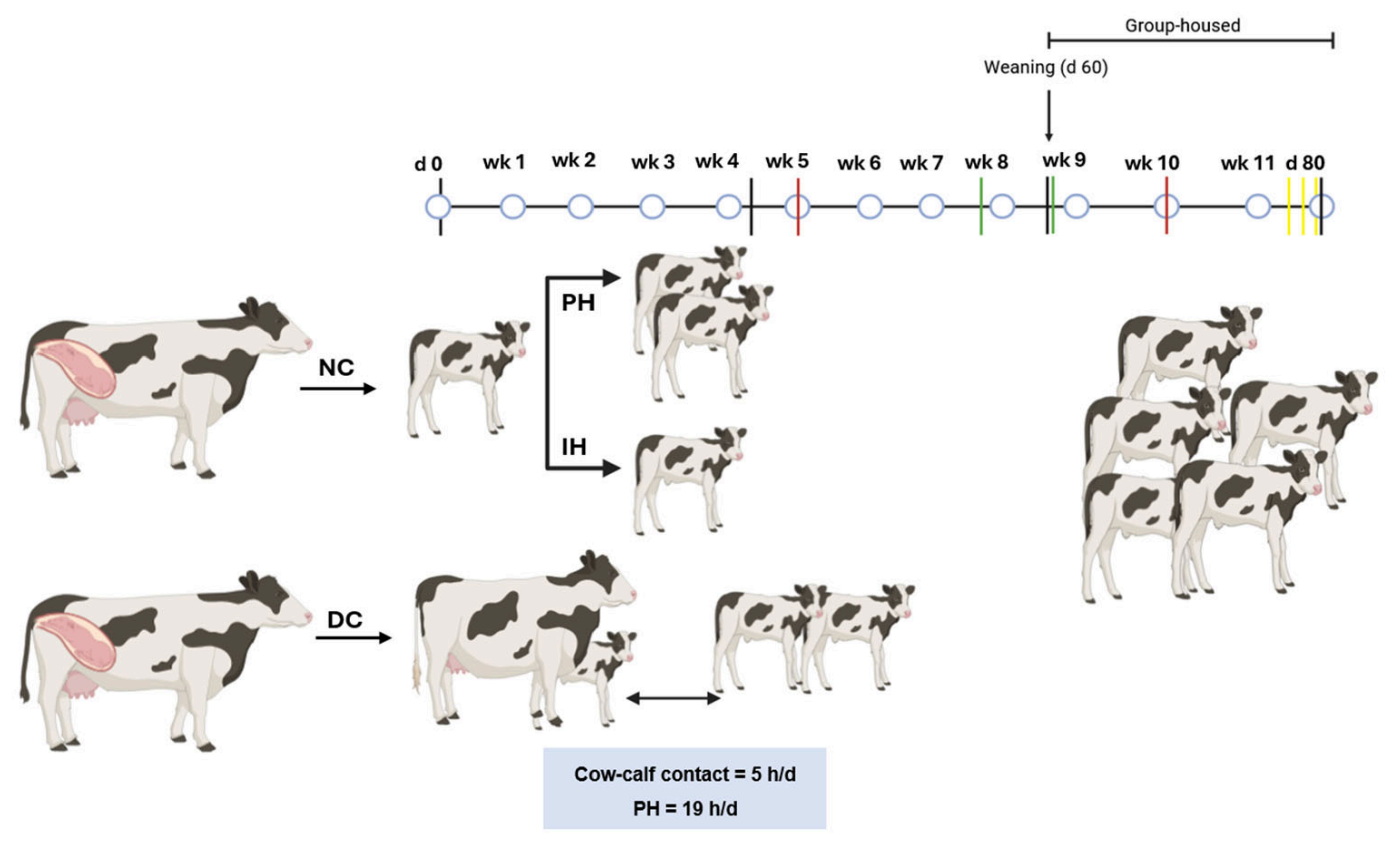
Timeline of experimental events and description of treatment groups. Arrows indicate the calf management treatments: DC-PH = dam contact (5 h/day), pair-housed; NC-IH = no dam contact, individually housed; NC-PH = no dam contact, pair-housed. After weaning, all calves were moved to group housing until day 80. The central timeline shows the schedule of animal-based measures: body weight (black; days 0, 30, 60, 80), skeletal growth (black; days 0, 30, 60, and 80), blood samples (green; days 54, 61), rumen fluid (red; days 35 and 70), fecal samples (yellow; days 78–80). Behavioral observations were conducted weekly from weeks 1 to 11 using 8-h observation periods and are represented on the timeline without color coding. The figure was created using BioRender (https://biorender.com).

Thirty newborn calves (19 males and 11 females; mean body weight = 37.5 ± 3.4 kg) were enrolled at birth and assigned to two treatment groups. Fourteen calves (8 males and 6 females) received 5 h/d of dam contact and were subsequently pair-housed (DC-PH). The remaining 16 calves were separated from their dam and assigned to the no-contact (NC) group, which was further subdivided into two subgroups: 8 calves were individually housed (NC-IH; 5 males and 3 females) and 8 were pair-housed (NC-PH; 4 pairs; 5 males and 3 females). Calves were allocated to treatment groups using a stratified randomization procedure based on birth body weight to achieve comparable groups. To minimize potential maternal effects, cows of consecutive parity were distributed evenly among treatment groups. No significant difference was observed in the 305-day milk yield of dams assigned to the DC-PH and NC groups (11,222.6 ± 1857.7 vs. 11,152.2 ± 1537.8 kg; *p* = 0.88). For DC-PH calves, dam contact was maintained until day 60, at which point all calves were weaned. All calves remained under observation until day 80. A visual representation of the experimental timeline and treatment groups is provided in Figure 1.

### 2.2. Housing and Management

All calves were initially housed in individual straw-bedded boxes in a naturally ventilated indoor barn until day 3 of age. Immediately after birth, NC calves were separated from their dams within 30 min, whereas DC-PH calves remained with their dams for 5 h in free-stall pens (4.7 × 3.15 m). From day 3, calves were moved to outdoor pens (2.1 × 2.2 m), either individually or with a peer. Pens were bedded with sawdust, which was replaced daily. After weaning (day 60), calves were moved to group housing (5 m wide × 8 m long) until day 80. Each pen contained 10 calves and was equipped with a water trough (0.30 m wide × 1.50 m long × 0.35 m high) and a feed bunk (0.40 m wide × 4.0 m long × 0.45 m high) to provide *ad libitum* access to water and solid feed.

### 2.3. Feeding Regimen

All calves were bottle-fed colostrum from their dam for the first two feedings, with volumes adjusted according to birth body weight (5%). This management strategy was implemented to ensure a standardized, timely, and quantifiable colostrum supply during the immediate postnatal period, when adequate colostrum intake is critical for the acquisition of passive immunity [21]. This also minimized the uncertainty associated with natural colostrum intake at the udder, where the quantity consumed by the calf is difficult to monitor and verify in CCC systems [6]. Colostrum quality and calf IgG levels were not evaluated. Following the second feeding, DC-PH calves were allowed to suckle any remaining colostrum directly from their dam. For NC calves, colostrum was followed by transition milk, with feeding adjusted according to their body weight during the first 3 days of life.

During the preweaning period, NC calves were fed saleable milk twice daily (0800 and 1600 h). Milk allowances were: 4 L/d (days 4–14), 5 L/d (days 15–28), 6 L/d (days 29–42), 7 L/d (days 43–53), and 4 L/d (days 54–59). For NC calves, the milk allowance was reduced to one meal daily during the final 6 days before weaning. In contrast, DC-PH calves suckled freely from their dams during the daily contact period (0900–1400 h). During this time, cow–calf pairs remained in the same free-stall pens as the dams, allowing animal contact. After the contact period, DC-PH calves were moved to the indoor barn, and no supplemental milk was provided. For DC-PH calves, contact time was gradually restricted from day 54 to 59 (3 h/d on days 54–55; 2 h/d on days 56–57; and 1 h/d on days 58–59). From day 3 onward, all calves had *ad libitum* access to water and starter feed. Cows were milked three times daily (0800, 1600, and 2400 h).

### 2.4. Measurements and Sampling

Ambient air temperature in the calf-rearing facility was continuously monitored using a thermometer and cross-referenced with regional meteorological data. From day 4 to 80, calves were clinically evaluated three times per week using a standardized health scoring system [22]. This system assessed respiratory signs, fecal consistency, rectal temperature, and navel inflammation on a 4-point scale (0 = normal, 3 = severe abnormality). Based on these evaluations, specific health conditions were identified using both composite and dichotomous classifications. Respiratory disease was defined by a composite respiratory score ≥ 4 (the sum of ocular discharge, nasal discharge, and cough scores). Neonatal diarrhea was characterized by a fecal score ≥ 2 (this category was included for the first 4 weeks of life as indication of neonatal diarrhea, and comprised either infectious diarrhea or feeding-related loose/liquid feces). Navel inflammation and fever were defined as navel scores ≥ 2 and rectal temperature scores of 3, respectively. All these variables were subsequently converted into dichotomous (presence/absence) categories for statistical analysis. Additionally, farm staff maintained a daily logbook to record all observed health issues and medical interventions, including antibiotic administrations, for both cows and calves. Calves requiring therapeutic treatment remained in the study and were included in all health and mortality analyses. Health events, therapeutic interventions, and mortality were recorded until death or the completion of the study. To provide a holistic measure of calf health, a Total Health Score (THS) was calculated, adapted from the approach described by van Dixhoorn et al. [23]. The THS represented the cumulative sum of all individual clinical scores recorded for each calf, from week 1 through 11. This metric integrated both the severity and duration of clinical symptoms; thus, lower THS values indicated fewer or shorter-lasting health issues, while higher values reflected more frequent or prolonged clinical illness.

The determination of feed intake involved daily weighing of the starter feed offered and refused before weaning, with values recorded at weekly intervals. Post-weaning feed intake was not quantified as calves were housed in group after weaning. Table 1 reported ingredients and chemical compositions of the starter diet fed to calves. During the experimental period, feed samples were collected daily, pooled weekly, and analyzed for nutrient composition. Dry matter content was determined by drying samples at 65 °C for 48 h in a forced-air oven, after which they were ground through a 1-mm screen using a Wiley mill (Arthur H. Thomas Co., Philadelphia, PA, USA), for the chemical analysis. Crude protein, crude ash, and ether extract were analyzed according to AOAC [24] methods, and ADF and NDF were determined following the methods described by Van Soest et al. [25].

For calves with dam contact, milk intake was estimated using the weigh-suckle-weigh procedure by measuring calf body weight immediately before and after the daily contact period with the dam [26]. However, because calves in the present study remained with the dam for 5 h, the estimated milk intake may have been affected by urine and fecal excretion during the prolonged contact period. Accordingly, milk intake values should be interpreted as approximate estimates rather than exact measurements of suckled milk consumption.

Calves were weighed at birth and on days 30, 60, and 80 using a calibrated scale (model EES-500; Etihad Co., Isfahan, Iran). Skeletal growth measures were assessed at birth and on days 60 and 80 following the methods described by Khan et al. [27]. Measurements included body length (distance between the points of the shoulder and rump), heart girth (chest circumference), hip width (distance between hook bones), withers height (distance from the front feet base to the withers), body barrel (belly circumference before feeding), chest width (distance between front feet), and hip height (distance from the rear feet base to the hook bones). Average daily gain (ADG) was calculated at the individual level as the difference in BW between successive measurement periods, divided by the number of days in each interval. Feed efficiency was calculated as the ratio of ADG (kg) to total DMI (kg).

Blood samples (5 mL) were collected from the jugular vein on day 54 (pre-weaning) and day 61 (post-weaning) using vacutainer tubes without anticoagulant. The samples were centrifuged at 2000 rpm for 20 min, and the serum was stored at −20 °C until analysis. Serum was analyzed for glucose (Delta Darman Part; Tehran, Iran), *β*-hydroxybutyrate (βHBA; Randox Laboratories Ltd., Crumlin, UK), and cortisol concentrations (Monobind Inc., Lake Forest, CA, USA).

To assess protozoal populations, rumen fluid (20 mL) was collected via an esophageal tube 3 h after the morning meal on days 35 and 70. The samples were strained through cheesecloth and fixed in aldehyde solution by mixing equal volumes of rumen fluid and 10% formalin. Protozoa were enumerated using a specialized counting slide under light microscopy at 40× magnification, and the results were expressed as concentration (number/mL of rumen fluid; [28]).

To determine apparent total-tract digestibility, fecal grab samples were collected from each calf twice daily for 3 consecutive days (d 78–80). The samples were frozen at −20 °C until analysis and then dried in a forced-air oven at 60 °C for 72 h. Acid-insoluble ash served as the internal marker for nutrient digestibility determination according to the method described by Van Keulen and Young [29].

Behavioral data were collected through direct observation of all calves, using definitions adapted from those presented in Table 2 [8,30]. Observations were conducted once weekly for an 8-h period by 4 trained observers. Observers were assigned to specific calves and were blinded to treatment allocation throughout the study. To verify observer accuracy, calf behavior was additionally recorded using a smartphone for several hours and compared with the corresponding direct observational records. Inter-observer reliability was not formally quantified. All observers followed a standardized ethogram comprising 10 predefined behavioral codes and adhered to a consistent observation protocol. Behaviors were recorded using instantaneous scan sampling at 1-min intervals, and the occurrence of each behavior was documented at each scan point (Figure 1). Thus, behavioral data represented the frequency of occurrence (i.e., the number of scans in which a behavior was observed) rather than the duration of each behavior. Data were summarized as mean frequencies across the observation period. A total of 10 behavioral observations (8-h sessions) were collected per calf during the study.

### 2.5. Statistical Analysis

Before analysis, data were assessed for normality using the UNIVARIATE procedure of SAS. Non-normally distributed non-nutritive oral behavior data were log-transformed prior to analysis. All statistical analyses were conducted using SAS 9.4 (SAS Institute Inc., Cary, NC, USA). Daily starter feed intake data were averaged by week before analysis.

Performance, metabolic, ruminal, and behavioral data were analyzed using repeated-measures mixed models. The models included treatment group, time, and the treatment × time interaction as fixed effects, with calf included as a random effect. Repeated observations within calf were modeled using a first-order autoregressive [AR(1)] covariance structure, which was selected based on the lowest Akaike Information Criterion (AIC) compared with alternative covariance structures, including Toeplitz and compound symmetry. Covariates, such as initial body weight and structural growth variables, were included when appropriate for the respective outcomes. Pairwise comparisons among treatment means were performed using Tukey’s test, with statistical significance declared at *p* ≤ 0.05 and tendencies at 0.05 < *p* < 0.10.

Nutrient digestibility data and THS were analyzed using a similar mixed model, excluding the time effect, as these were analyzed as overall means per calf. Health status was recorded three times weekly as a binary outcome. For each calf, the number of positive observations was summed across the 33 evaluation periods to generate a calf-level health score. Because of the low prevalence of positive events and the presence of zero counts in some categories, clinical symptoms of specific health variables and the average of calves treated with antibiotics were compared among treatment groups using Fisher’s exact test. Three calves died before 1.5 months of age. Data from these animals were treated as missing after mortality and were analyzed using the mixed model based on all available observations collected prior to death.

**Table 1:** Ingredients and chemical compositions of the starter diet fed to calves.

Ingredient (% of DM)	Value	Chemical Composition (% of DM)	Value
Barley grain	10	DM, % of as fed	92.8
Corn grain	47.1	CP	19.8
Soybean meal (45% CP)	33	Ether extract	2.47
Wheat straw	4.0	NDF	19.5
Dicalcium phosphate	0.5	Ash	8.11
Sodium bicarbonate	1.0	ME ^2^ (Mcal/kg of DM)	3.0
Salt	1.4		
Calcium carbonate	1.5		
Vitamin premix ^1^	1.5		

^1^ Contained per kg of supplement, 1,200,000 IU vitamin A, 250,000 IU vitamin D, 10,000 IU vitamin E, 8.5 g Mn, 320 g Ca, 11.5 g Zn, 5 g Mg, 105 mg Co, 3.7 g Cu, 190 mg I, and 105 mg Se. ^2^ Calculated according to NRC [31].

**Table 2:** Description of the recorded behavioral activities *.

Behavior	Definition
Lying	Lying on sternum with the head either raised or placed down.
Standing	Upright posture with all four feet on the ground, either inactive or active.
Eating	Consumption of starter feed from the bunk or milk, including when the head is positioned inside the bunk.
Ruminating	Irregular, repetitive chewing without discernible food in the mouth, occurring either lying or standing.
Self-grooming	Movements with tongue over own body surface or scratching with a leg.
Non-nutritive behavior	Biting, sucking or licking any pen structures, except for feed; consuming bedding materials, and calf rolling its tongue outside the mouth.
Social contact	Licking, sniffing, or gently biting another calf.
Walking	Moving at least two legs to change body position in a forward, backward or sideways motion.
Vocalizations	All types of vocalizations.
Solitary play	Locomotor activity such as leaping, jumping, bucking, or turning.

* [8,30].

## 3. Results

### 3.1. Environmental Conditions

**Figure 2:**
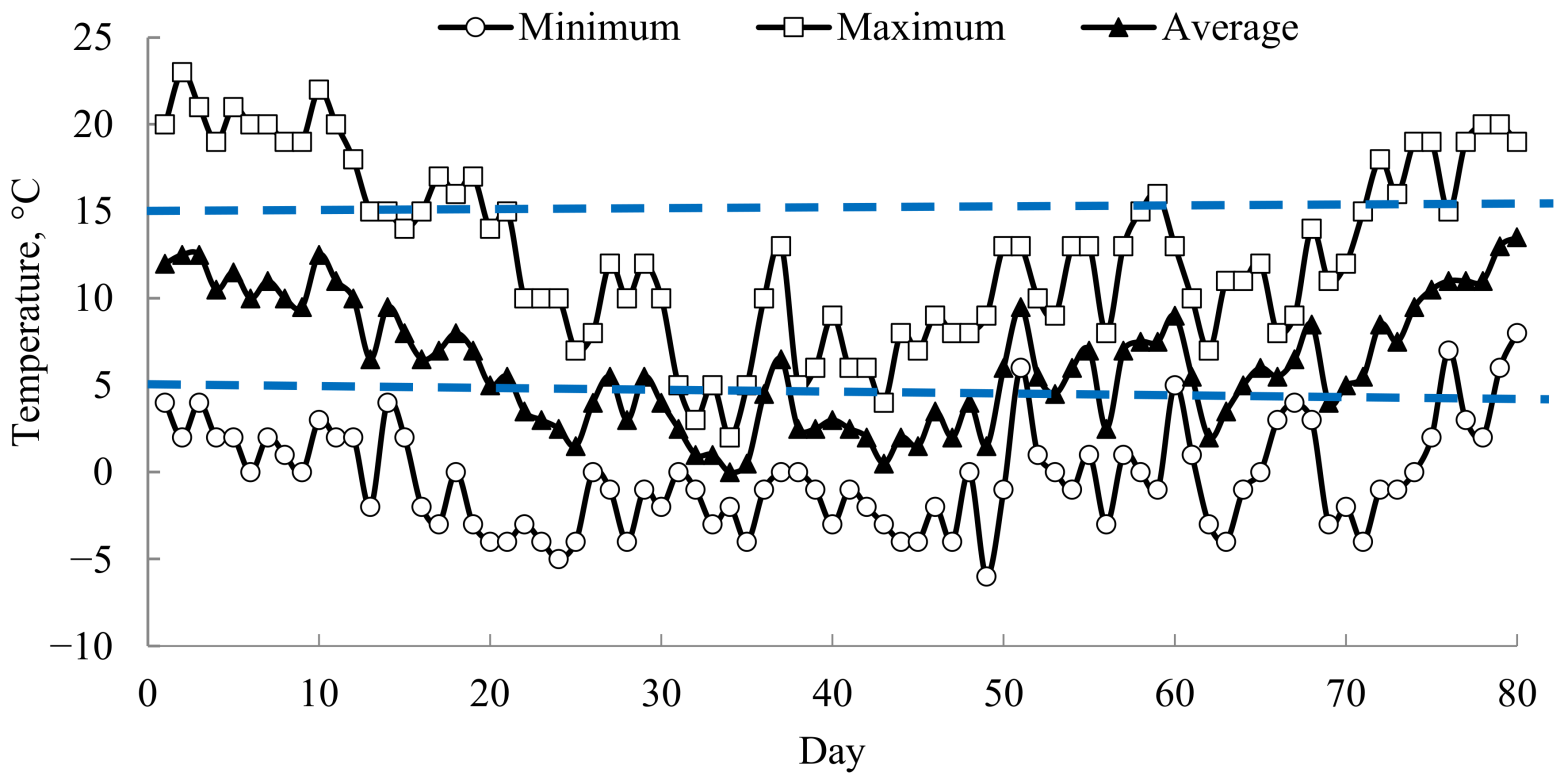
Pattern of daily temperature changes (minimum, average and maximum) recorded during the 80-d experimental period. Dashed lines represent the lower critical temperature thresholds for calves ≤ 3 wk of age (15 °C) and >3 wk of age (5 °C).

Daily air temperature profile in the calf rearing facility is presented in Figure 2; mean maximum, average, and minimum values during the trial were 12.7, 6.2, and −0.3 °C, respectively. Air temperature in the free-stall barn used for dam-calf contact was similar to that of the calf-rearing facility. However, DC calves were additionally exposed to environmental conditions while moving through an uncovered passage to and from the free-stall area. Moreover, the free-stall barn was more exposed to wind, potentially increasing the calves’ exposure to climatic stressors.

### 3.2. Health of Calves and Antibiotic Use

The effects of dam contact and social grouping on THS are presented in Table 3, and the prevalence of diseases is summarized in Table 4. Approximately 90% of clinical symptoms were observed during the first 7 weeks of the study, after which the calves remained in relatively good health. DC-PH calves tended (*p* = 0.06) to have a higher mean cumulative THS (4.14 ± 1.08) compared with NC-PH calves. Within the NC group, NC-IH calves had a significantly higher THS (3.63 ± 1.05) than NC-PH calves (1.13 ± 0.44; *p* = 0.05). During the initial 7 weeks, 37% of the calves required antibiotic treatment, with no significant differences in antibiotic usage observed among the treatment groups. During the trial, three calves succumbed to severe pneumonia (two from the DC-PH group and one from the NC-IH group). No significant differences in disease prevalence were observed among the treatments (*p* > 0.05; Table 4).

### 3.3. Performance Outcomes

The effects of treatment and time on calf performance are summarized in Table 5. There were no significant differences in body weight at birth among the groups; however, a significant time × treatment interaction was observed (*p* = 0.01), with DC-PH calves achieving a significantly higher body weight by day 80 compared to NC-PH calves (92.8 vs. 83.8 kg; *p* = 0.01). Calves in DC-PH group exhibited an increase in ADG from days 60–80 (1.332 kg) compared to the NC-PH group (0.898 kg). No significant growth differences were observed between NC-IH and NC-PH calves. Pre-weaning solid feed intake and feed efficiency did not differ among treatment groups, and no treatment × time interaction was observed for these parameters (*p* > 0.10). As expected, milk intake was significantly higher in DC-PH calves compared with NC-PH calves. The total pre-weaning milk dry matter intake of DC-PH calves averaged 55.4 kg ± 8.4 kg/calf, with a coefficient of variation of 15.2% among individual calves.

The effects of dam contact and social housing on skeletal growth measures are presented in Table 6. Hip width, body barrel, and hip height did not differ significantly across treatment groups, but significant time × treatment interactions were observed for body length (*p* < 0.01), withers height (*p* = 0.02), and heart girth (*p* = 0.03). Calves in DC-PH group had a significantly greater growth between day 60 and day 80 compared to the NC-PH group. Consequently, on day 80, DC-PH calves had greater body length compared with NC-PH calves (*p* = 0.05). Furthermore, withers height and chest width showed a tendency toward significance in DC-PH calves compared with NC-PH calves (*p* = 0.08 and *p* = 0.09, respectively). No significant differences were observed between NC-IH and NC-PH calves in any of calf skeletal growth measures.

### 3.4. Blood Metabolites

The effects of dam contact and social housing on selected blood metabolites and protozoa counts in the calf rumen are presented in Table 7. Blood glucose on day 54 was greater in DC-PH than NC-PH calves (*p* = 0.05). There was no significant time × treatment interaction (*p* = 0.66). In contrast, a significant interaction was detected for βHBA (*p* < 0.01), where circulating βHBA concentration was lower before weaning but higher after weaning in DC-PH vs. NC-PH calves, with a tendency towards significance (*p* = 0.06 and *p* = 0.09, respectively). DC-PH calves had a lower cortisol concentration on day 54 compared to NC-PH calves (*p* = 0.02). No significant differences in blood glucose, βHBA, or cortisol were observed between NC-IH and NC-PH calves.

Calves in DC-PH group had a significantly higher proliferation of protozoa on day 35 and 70 compared to those in NC-PH group (*p* = 0.01 and *p* < 0.01, respectively). On day 35, protozoa abundance did not differ between NC-IH and NC-PH calves. In contrast, on day 70, protozoa counts were greater in NC-PH calves compared with NC-IH calves (*p* = 0.04).

### 3.5. Nutrient Digestibility

The effects of dam contact and social housing on DM and NDF digestibility are shown in Table 8. No significant differences in DM and NDF digestibility were observed between DC-PH and NC-PH calves. However, within the NC groups, NC-PH calves had greater DM digestibility compared to NC-IH calves (87.7 vs. 81.4; *p* = 0.03).

### 3.6. General Behavioral Patterns

**Figure 3:**
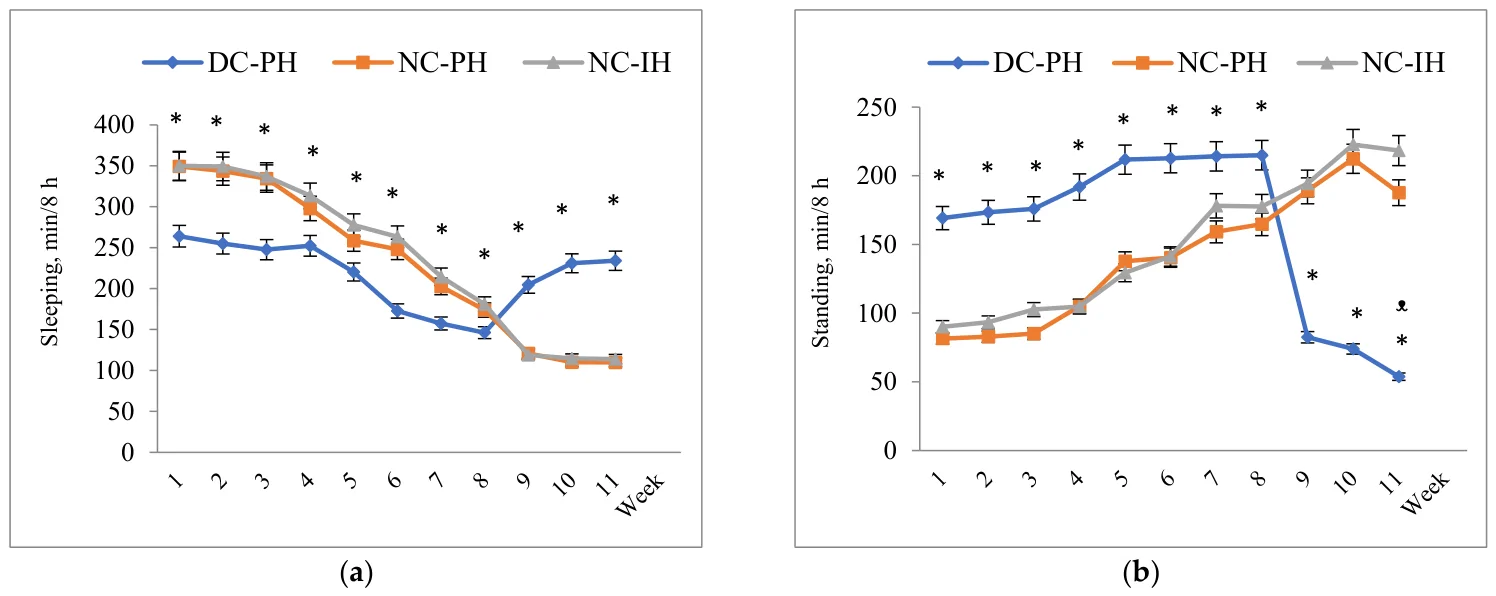

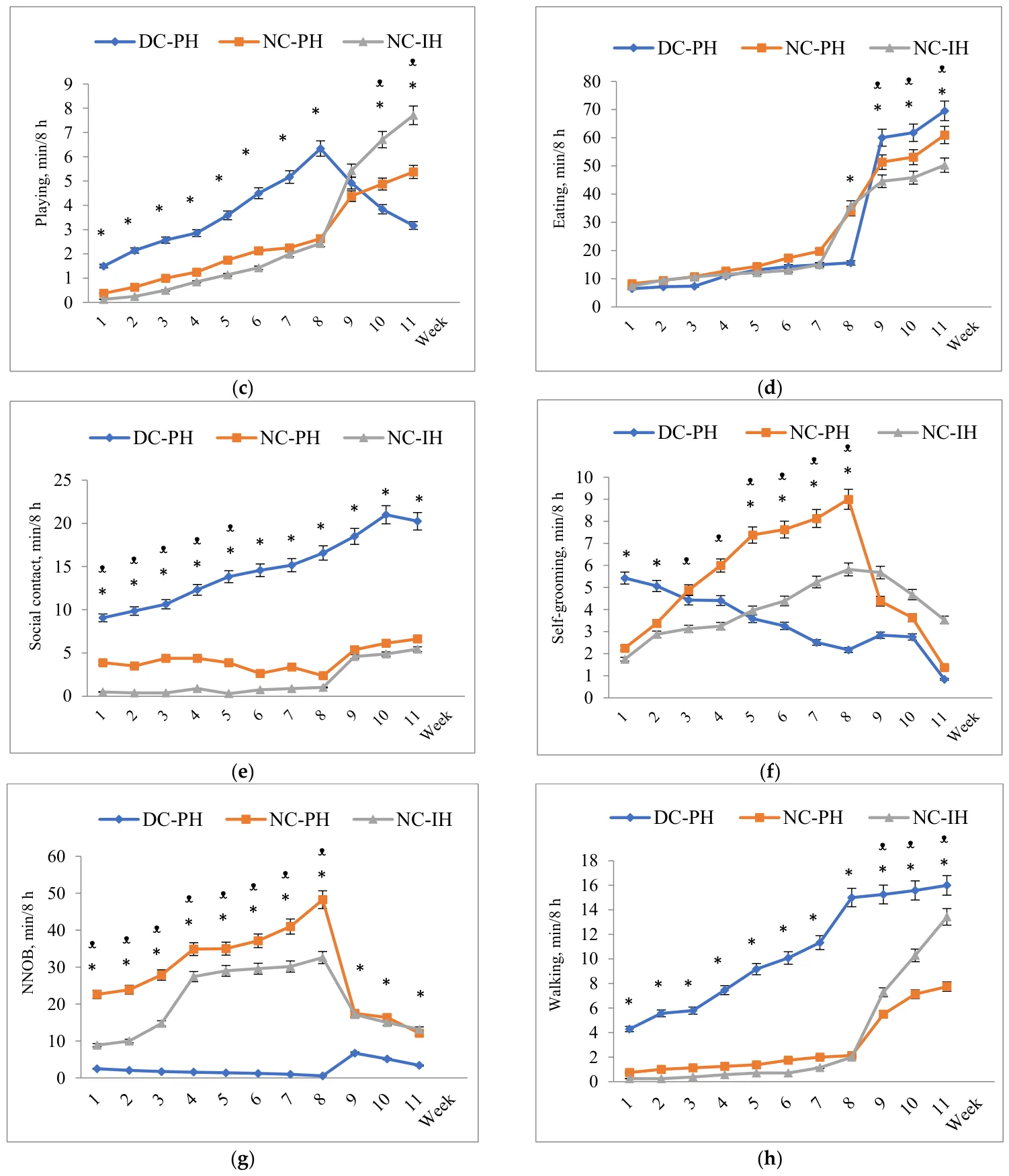

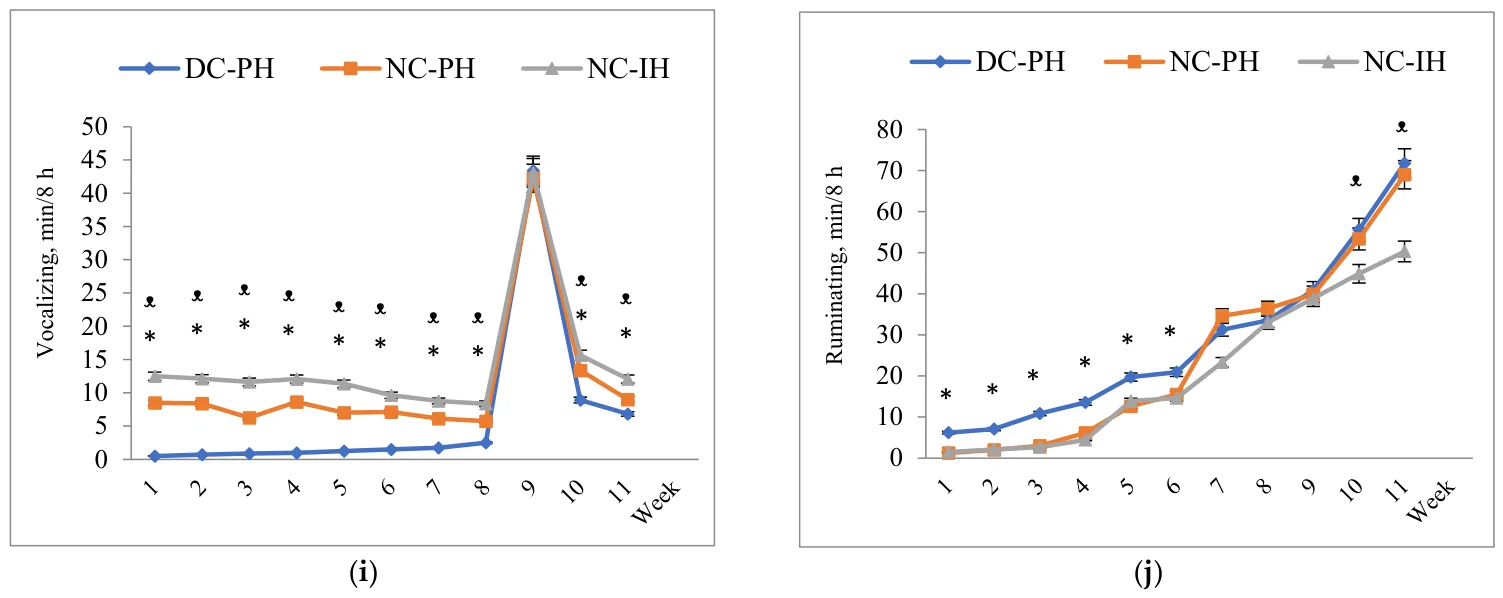
Interaction effects of treatment group (DC-PH = 5 h/d dam contact, pair-housed; NC-IH = no dam contact, individually housed; NC-PH = no dam contact, pair-housed) and time on calf behaviors during the pre-weaning (weeks 1–8) and post-weaning (weeks 9–11) periods. Weaning occurred on day 60. Behaviors shown are (**a**) sleeping, (**b**) standing, (**c**) playing, (**d**) eating, (**e**) social contact, (**f**) self-grooming, (**g**) non-nutritive oral behaviors (NNOB), (**h**) walking, (**i**) vocalizing, and (**j**) ruminating. An asterisk (*) indicates a significant difference between DC-PH and NC-PH calves. The symbol (ᵜ) indicates a significant difference between NC-PH and NC-IH calves (*p* < 0.05).

The effects of dam contact and social housing on calf behaviors are presented in Figure 3. During the pre-weaning period, DC-PH calves were observed standing more frequently than NC-PH calves; however, this trend reversed after weaning, as NC-PH calves were observed standing more frequently than DC-PH calves. Except for week 11 (*p* < 0.01), no differences were observed between NC-IH and NC-PH calves in standing behavior. Similarly, DC-PH calves were observed resting (sleeping) less frequently than NC-PH calves before weaning but exhibited the highest frequency of resting observations post-weaning (*p* < 0.01). No differences in resting observations were detected between NC-IH and NC-PH calves. Play behaviors (leaping and jumping) were more frequent in DC-PH calves during the milk-feeding period but decreased after weaning, at which time NC calves (particularly NC-IH) exhibited greater play activity (*p* ≤ 0.05). The frequency of eating observations did not differ between treatments until week 7. At week 8, the frequency of eating observations was greater in NC-PH than DC-PH, whereas post-weaning (weeks 9–11), it was greater in DC-PH calves (*p* < 0.01). Within the NC group, no differences were observed pre-weaning, but post-weaning, NC-PH calves were observed eating more frequently than NC-IH calves (*p* = 0.03). Calves in the DC-PH group were consistently observed engaging more frequently in social contact and walking behaviors than NC-PH calves (*p* < 0.01). Within the NC group, NC-IH calves were observed walking more frequently post-weaning than NC-PH calves. Only from weeks 1–5, NC-PH calves were observed engaging more frequently in social contact than NC-IP calves. Self-grooming observations were initially more frequent in DC-PH calves during the first 2 weeks but declined thereafter; conversely, NC-PH calves showed increasing self-grooming observations until week 8 and were greater than NC-IH calves. After week 8, self-grooming frequency declined in both groups, with no significant difference observed between NC-PH and NC-IH calves.

Non-nutritive oral behaviors (e.g., licking and cross-sucking) were observed least frequently in DC-PH calves and most frequently in NC-PH calves throughout the 11-week period (*p* < 0.01). Before weaning, these behaviors were observed more frequently in NC-PH than in NC-IH calves (*p* < 0.01), but no differences were observed post-weaning. Vocalization was least frequent in DC-PH calves and most frequent in NC-IH calves by week 8. During week 9 (the first week post-weaning), vocalizations increased sharply across all groups with no treatment differences across groups (*p* = 0.10). Thereafter, vocalizations declined, remaining least frequent in DC-PH calves (*p* < 0.05). DC-PH calves were observed ruminating more frequently than NC-PH calves up to week 6, with no differences observed thereafter (weeks 7–11). Post-weaning (weeks 10 and 11), NC-PH calves were observed ruminating more frequently than NC-IH calves (*p* < 0.01).

## 4. Discussion

An important consideration when interpreting the present findings is that the experimental design compared three practical calf-rearing management systems rather than a complete 2 × 2 factorial arrangement. Thus, the main effects of dam contact and social housing, as well as their interaction, could not be estimated. Therefore, the influence of partial dam contact was discussed primarily from comparisons between the DC-PH and NC-PH treatments; the effects of housing are reflected by comparisons between the NC-PH and NC-IH treatments.

Exposure to cold stress increases calf energy requirements for thermoregulation, thereby elevating maintenance costs and reducing energy available for growth if not compensated by increased nutrient intake [16,32]. Holt [32] reported that winter-born calves consumed more starter feed but achieved similar weight gains, indicating that dietary energy was diverted toward heat production rather than tissue accretion. In this experiment, pre-weaning feed intake (except during days 0–30) and growth performance did not differ among treatment groups. This is likely reflective of increased energy expenditure for thermoregulation. Calves with dam contact consumed less starter feed during the first month of life (d 0–30), most likely because their greater milk intake through suckling met their nutrient requirements and reduced the motivation to consume solid feed. Previous research has shown that calves fed large quantities of milk generally consume less solid feed pre-weaning [12,33] and that full dam contact calves typically ingest less starter than limit-fed artificially reared calves [34,35]. A review by Khan et al. [36] also concluded that higher milk allowances decreased pre-weaning solid feed intake likely because of inducing satiety through both metabolic feedback (increased circulating glucose and insulin level) and physical gut fill from abomasal curd, thereby reducing the calf motivation to consume solid feed. From days 30–60, starter feed intake of DC calves was not different from NC calves, despite the higher milk intake in DC calves. This may suggest that as calves mature, factors such as increased rumen capacity and the developmental changes in feeding motivation may affect the satiety-related suppression of solid feed intake observed earlier in the pre-weaning period.

Most studies on calves reared in CCC systems have reported a growth advantage compared with non-contact calves, although some studies have found reduced or no effects (e.g., increased growth [4] or decreased growth [11]). Consistent with the present study, Froberg et al. [37] found no difference in weight gain between treatments, although they reported greater variability within the suckling group. However, we observed increased growth in DC calves after weaning (day 80). This finding aligns with Meagher et al. [38], who reported that growth benefits of dam contact persisted for several months post-weaning, but contrasts with Wenker et al. [39], who observed no post-weaning differences in body weight across separation strategies. One possible explanation is that social facilitation and social learning may promote earlier and greater solid feed intake in group-housed calves [4]. The post-weaning growth advantage observed in DC calves may therefore reflect enhanced social skills, improved resilience to weaning stress, reduced neophobia, and facilitated adaptation to post-weaning feeding conditions via social interactions. However, post-weaning feed intake was not measured in the present study.

High milk allowances during early life are known to reduce starter intake and may delay rumen epithelial development and fibrolytic activity by limiting solid feed consumption and ruminal fermentation stimulation [36,40]. Consequently, calves receiving greater quantities of milk often exhibit reduced fiber digestion during the early life. However, previous studies have demonstrated that when calves are weaned at approximately 50 to 60 days of age, rumen adaptation and starter intake generally become sufficient to support effective post-weaning digestion regardless of pre-weaning milk allowance [41,42,43]. In the present study, weaning on day 60 likely provided adequate time for compensatory rumen development in DC calves despite their greater milk consumption. However, the increased dry matter digestibility in pair-housed calves (NC-PH) compared to individually housed calves (NC-IH) may suggest that social environment plays a crucial role in modulating rumen development and function.

Ruminal protozoa contribute substantially to fiber degradation, particularly hemicellulose digestion, and may account for 25–30% of total ruminal fiber digestion [44]. However, despite the greater protozoal abundance in DC-PH calves than in NC-PH calves, this difference was not accompanied by a significant increase in NDF digestibility. The dam has long been hypothesized to serve as the primary source of microbial inoculation for the calf rumen [45]. Beyond providing nutritional support, maternal contact may therefore be essential for the early establishment of a diverse ruminal microbiota. In artificially reared calves, separation from the dam and reliance on milk replacer are thought to limit microbial exposure, particularly hindering the development of ciliate protozoa [46]. Increased starter intake is recognized as a key driver of microbial proliferation in the developing rumen [43,47]. In the present study, solid feed intake did not differ among treatment groups; nevertheless, protozoal counts were consistently greater in DC-PH calves than in NC-PH calves. This indicates that dam contact itself, rather than solid intake, was the main contributor to enhanced protozoal colonization. Direct microbial transfer from the dam via saliva, skin, or fecal contact may therefore have facilitated protozoal establishment in DC calves.

In neonatal calves, circulating glucose concentration is relatively high because energy supply is derived primarily from intestinal lactose absorption before rumen function is fully established [48]. With advancing age, glucose concentrations decline as energy metabolism shifts toward volatile fatty acids produced via ruminal fermentation [49]. In our study, the higher pre-weaning glucose concentrations observed in DC calves likely reflected their greater milk consumption and reduced ruminal development relative to NC calves, consistent with findings that lactose digestion provides the major energy source during this phase [49,50].

Blood circulating βHBA is widely considered as a biomarker of functional rumen development in pre-weaned and weaned calves, as its concentration rises with increasing intake of starter feed and enhanced ruminal fermentation capacity [51]. The concentration of βHBA rises with increasing starter feed intake and enhanced ruminal fermentation capacity, as it is a key ketone body synthesized by the rumen epithelial tissue following the absorption and metabolism of butyrate [51,52].

Perez et al. [53] demonstrated that separating calves from their mothers stimulates stress responses in both, including vocal behavior, greater locomotor activity, fence-line seeking, reduced body weight, and higher cortisol concentrations. Cortisol, in particular, is widely recognized as a reliable indicator of stress and welfare status [53]. Before weaning, DC-PH calves had a lower cortisol concentration than NC-PH calves, suggesting reduced stress and potentially improved welfare under dam contact conditions.

Prolonged CCC has been shown to influence both physical and behavioral development [39]. In the present study, calves with half-day dam contact were more frequently observed standing, playing, walking, and engaging in social interactions with peers before weaning than NC-PH calves. After weaning and integration, DC-PH calves were more frequently observed resting and eating than NC-PH calves, consistent with the advantages of CCC in reducing aggression and promoting adaptation to the environment. Similar patterns have been reported in heifers reared with full CCC, which tended to be more submissive than heifers reared without CCC [54,55]. These outcomes suggest smoother adjustment to group housing and are consistent with previous studies reporting shorter lying latencies, longer feeding durations, and more frequent lying bouts in CCC-reared animals [54]. Also, as dairy calves are highly motivated for early-life social contact [56], these behavioral advantages highlight CCC as an important welfare factor.

Greater frequency of standing observations during pre-weaning period in DC-PH calves (as compared to NC-PH) might indicate a strong motivation to interact with their dams, supported by literature suggesting reduced exploratory behavior in the absence of dam [1]. Conversely, a wider and more active range of social behaviors during conspecific interactions (such as head butting, sniffing, tail wagging, rubbing, and play) has been observed in calves reared with CCC relative to those reared without CCC [57,58]. This agrees with our findings as DC-PH calves expressed a wider range of social behaviors than NC-PH calves both prior to weaning and after integration.

Full CCC has been shown to reduce abnormal behaviors in calves. In addition to a decrease in tongue rolling [59,60], studies have reported a rare occurrence of cross-sucking and non-nutritive sucking in full CCC rearing systems [61,62], although some studies found no differences [11]. Natural suckling most likely accounts for these effects as it facilitates a higher milk consumption (~9 L/d during 9 wk of part-time contact [63] and ~15 L/d during 13 wk of full contact [62]), meeting the calf’s need to suckle, and allowing more frequent milk meals [64]. In contrast, common artificial feeding practices often rely on buckets or teat buckets that deliver milk at a high flow rate, offering calves little opportunity to satisfy their suckling motivation, resulting in limited or absent oral stimulation [65]. In this experiment, the occurrence of non-nutritive behavior such as cross-sucking of other calves or sucking of fences and objects was lower in DC-PH than in NC-PH calves throughout the experimental period. It is likely that NC-PH calves may have redirected their unsatisfied suckling motivation toward non-nutritive oral behaviors, which could explain the greater occurrence of these behaviors in this group.

Play is widely recognized as beneficial for animal welfare; however, restricted social environments during early development may constrain both the opportunity and the motivation to engage in play [4]. Increased space allowance promotes play behavior by minimizing environmental restrictions and enhancing opportunities for play. In this experiment, NC-PH calves showed more solitary play during the integration period (after weaning) compared to DC-PH calves. This increase in solitary play likely represents a behaviour rebound triggered by the larger test arena compared with the confined home pen [4]. These findings agree with the findings of Wenker et al. [39] and Wagner et al. [58], who reported that calves reared without CCC engaged in more solitary play during post-integration tests compared with calves reared with full CCC. Calves with full CCC often perform locomotor play in the alleys of the cow barn [58,66]. This may reduce their motivation for locomotor play in the test arena relative to calves that experienced more restricted housing conditions [58]. In the present experiment, it is likely that the lower expression of play behavior in DC-PH calves after integration may reflect their greater opportunities to engage in locomotor play while with their dam before weaning; the greater play behavior of NC-PH calves after weaning may represent a rebound response following release from a more spatially restricted environment.

Calves frequently engage in self-grooming, which contributes to hygiene maintenance [67]. The sensitivity of this behavior to physiological and environmental influences suggests a possible connection to calf comfort and welfare. A reduction in self-grooming has been reported following experimental disease challenges designed to induce sickness behavior [68]. Self-grooming is a behavior that is associated with the comfort, welfare, health and maintaining the hygiene of an animal [67,68]. Greater occurrence of self-grooming during pre-weaning period in NC-PH than NC-IH calves may partly be related to housing conditions, as two NC-PH calves were housed in pens of the same size as those occupied by one NC-IH calf. Thus, NC-PH calves may have had greater contact with their manure, potentially increasing the need for self-grooming to maintain hygiene. Bertelsen and Jensen [14] stated that compared with artificial rearing, where calves have either no or very limited social contact (individual housing), or only calf contact (pair or group housing) during the milk feeding period, half-day contact still provides opportunity for suckling and maternal grooming. The reduced occurrence of self-grooming in DC-PH than NC-PH calves (weeks 5–8) may result from the behavior displayed by cows, which involves licking, nursing, staying close to the calf, and protecting it from threats [69]. Therefore, maternal licking can reduce the need for calves to self-groom. Once calves were weaned and integrated, the frequency of self-grooming showed a decreasing trend in all groups. This pattern may reflect a shift toward social grooming, which agrees with social contact behaviors and is thought to support the development of social bonds, as calves spend more time grooming familiar companions than unfamiliar ones [70].

Before weaning, DC-PH calves consistently exhibited lower vocalization than NC-PH calves; however, the first week after weaning (week 9), vocalization increased markedly and did not differ between the two groups. The sudden increase in vocalization observed in DC-PH calves after weaning (week 9) may reflect their motivation to reunite with the cow and obtain milk, rather than solely the need for milk [71,72]. Stressful challenges to animal welfare, such as weaning, commonly elicit distress behaviors including increased activity and high-pitched vocalizations [73,74]. The development of these distress behaviors can be interpreted as adaptive responses aimed at signaling a need for resources [75]. Because DC calves simultaneously endured maternal separation and milk withdrawal, the marked increase in their vocalization likely reflected the combined effects of these stressors. Overall, these findings suggest that maternal contact and social housing positively influenced behavioral development and reduced the expression of abnormal and stress-related behaviors, supporting improved welfare during the pre-weaning and weaning periods.

Cold environmental conditions during the study period likely contributed to the health challenges observed in the calves by increasing maintenance energy requirements and susceptibility to disease [16]. Housing conditions, particularly barn climate, bedding quality, and hygiene, may further influence calf vulnerability to diseases [39,76]. Studies on calf social behavior during winter have shown that physical contact among calves may reduce metabolic stress by decreasing heat loss and providing social facilitation [77]. Therefore, the higher prevalence of these disorders in NC-IH calves may be explained, at least in part, by their greater exposure to adverse weather conditions. In the present study, diarrhea and respiratory diseases, two of the most prevalent disorders affecting young calves [78], were identified as primary factors contributing to the increased THS. This increase may be attributed to higher exposure to adverse environmental conditions; as DC calves were moved to free-stall housing without adequate shelter, they were more susceptible to weather fluctuations. Furthermore, the free-stall locations were more exposed to wind gusts. The risk of respiratory disease is increased under suboptimal housing conditions, including inappropriate temperature, humidity, drafts, poor air quality, and wet bedding [79]. In contrast, inadequate hygiene, housing, and feeding management are recognized risk factors for diarrhea [80]. The elevated THS observed in DC calves was largely attributable to diarrhea, which may have been associated with excessive milk intake predisposing calves to digestive disturbances [62]. Because calves in the DC treatment voluntarily consumed more milk than calves in the NC treatments, the greater incidence of diarrhea, as well as differences in growth, metabolic status, and rumen development, may have reflected the combined effects of increased nutrient intake and maternal exposure rather than dam contact alone. As milk intake was not standardized across treatments, the independent effect of maternal contact cannot be distinguished from that of greater nutrient intake. In addition, group housing may have increased exposure to enteric pathogens through contact with contaminated surfaces [15]. These findings are consistent with previous studies reporting a higher incidence of diarrhea in calves reared with dam contact [39,62,81]. However, other studies evaluating CCC systems have reported neutral or even beneficial effects on calf health [62,74].

These inconsistencies may reflect differences in study design and methodology, as many of these studies did not assess calf health as a primary outcome, were conducted in facilities with differing barn designs, and often relied on relatively small sample sizes. Similarly, the relatively small number of calves enrolled in the present study, together with the loss of 3 calves during the experimental period, likely limited the statistical power to detect differences in relatively infrequent health outcomes. Thus, some clinically relevant differences among treatments may not have reached statistical significance, whereas statistically significant findings should also be interpreted with appropriate caution. Although three calf mortalities occurred during the study, the number of events was too small to allow a robust statistical evaluation of mortality as a separate outcome. A further limitation of this study is that microenvironmental conditions, including wind exposure and moisture, were not quantified separately in the outdoor calf pens and the free-stall contact area. Calves in DC-PH treatment were exposed to these conditions during daily transfers, but the exposure was limited to only short transition periods. The potential contribution of these unquantified environmental factors to the observed outcomes cannot be excluded, however, this brief exposure is unlikely to have contributed to the observed differences among treatments. Future studies should evaluate social companionship in housing systems with comparable environmental conditions to eliminate microenvironmental exposure as a potential confounding factor.

Farmers transitioning from conventional rearing to CCC systems have generally reported benefits for both calves and cows, although they also noted the need for additional infrastructure and management adjustments [82,83]. These practical considerations are consistent with the present findings, which suggest that partial dam contact, together with greater voluntary milk intake, may improve behavioral and developmental outcomes, but that successful implementation, particularly under cold-season conditions, requires careful attention to housing, hygiene, and environmental management to minimize disease risk. Future studies should include a control group receiving equivalent milk intake to isolate the effects of maternal contact from those of increased milk intake. In addition, studies with larger sample sizes are needed to provide sufficient statistical power to confirm the effects of these rearing systems on calf health and survivability.

## 5. Conclusions

Our findings indicated that the partial dam contact system (DC-PH), which was associated with greater voluntary milk intake, maintained similarly high levels of nutrient digestibility but tended to have an overall poorer health status, as compared to NC-PH group. The findings further suggested that partial dam contact was associated with a greater abundance of ruminal protozoa and likely differences in rumen development. Lower cortisol concentration and fewer abnormal behaviors were also observed in DC-PH than in NC-PH calves. However, as calves in DC-PH treatment also consumed more milk and milk intake was not standardized across treatment groups, these responses should be interpreted as reflecting the combined effects of maternal contact and increased nutrient intake, and thus the independent contribution of maternal contact cannot be distinguished. The presence of both a companion and the dam appeared to reduce behavioral signs of social isolation, which may be particularly important during the pre-weaning period when calves are physiologically vulnerable. These findings emphasize that the effects of partial dam contact on calf welfare are multifaceted, with favorable behavioral and physiological responses occurring alongside potential health trade-offs. Overall, partial dam contact system represents a promising rearing approach that supports both calf welfare and milk harvest efficiency, provided that management strategies effectively mitigate health risks and weaning stress. Future research should aim to optimize the duration and environmental management of CCC across different climatic and production systems to balance productivity, welfare, and societal expectations for sustainable dairy production.

## Figures and Tables

**Table 3:** Effect of dam contact and social companion on health (mean ± SE).

Items	Treatment Groups *			*p*-Value	
	NC-IH (n = 8)	NC-PH (n = 8)	DC-PH (n = 14)	NC-IH vs. NC-PH	DC-PH vs. NC-PH
Total health score	3.63 ± 1.05	1.13 ± 0.44	4.14 ± 1.08	0.05	0.06
Antibiotic use	0.38 ± 0.18	0.25 ± 0.16	0.43 ± 0.14	0.62	0.43

* NC-IH = no dam contact, individually housed; NC-PH = no dam contact, pair-housed; DC-PH = dam contact (5 h/d), pair-housed. Sample sizes represent the number of calves originally allocated to each treatment group. Three calves died during the trial (two in DC-PH and one in NC-IH); data collected before mortality were retained in the analyses, whereas observations after death were treated as missing in the repeated-measures mixed model.

**Table 4:** Effects of dam contact and social companion on prevalence of calves (%) that were classified at least once with clinical symptoms for various health variables (% ± SE; 95% CI).

Items	Treatment Groups *			*p*-Value	
	NC-IH (n = 8)	NC-PH (n = 8)	DC-PH (n = 14)	NC-IH vs. NC-PH	DC-PH vs. NC-PH
Nasal discharge	37.5 ± 0.17	12.5 ± 0.12	35.7 ± 0.13	0.57	0.35
	(0.09–0.76)	(0.003–0.53)	(0.13–0.65)		
Ocular discharge	25 ± 0.15	12.5 ± 0.12	28.6 ± 0.12	0.99	0.61
	(0.03–0.65)	(0.003–0.53)	(0.08–0.58)		
Cough	62.5 ± 0.17	25 ± 0.15	35.7 ± 0.13	0.31	0.99
	(0.24–0.91)	(0.03–0.65)	(0.13–0.65)		
Diarrhea	62.5 ± 0.17	37.5 ± 0.17	57.1 ± 0.13	0.62	0.66
	(0.24–0.91)	(0.09–0.76)	(0.29–0.82)		
Naval inflammation	0.0	12.5 ± 0.12	0.0	0.99	0.36
		(0.003–0.53)			
Fever	25.0 ± 0.15	12.5 ± 0.12	21.4 ± 0.11	0.99	0.99
	(0.03–0.65)	(0.003–0.53)	(0.05–0.51)		

* NC-IH = no dam contact, individually housed; NC-PH = no dam contact, pair-housed; DC-PH = dam contact (5 h/d), pair-housed. Sample sizes represent the number of calves originally allocated to each treatment group. Three calves died during the trial (two in DC-PH and one in NC-IH); data collected before mortality were retained in the analyses, whereas observations after death were treated as missing in the repeated-measures mixed model.

**Table 5:** Effect of dam contact and social companion on calf performance.

Items	Treatment Groups *			SEM	*p*-Values			
					Treatment		Time	Time × Treatment
	NC-IH (n = 8)	NC-PH (n = 8)	DC-PH (n = 14)		NC-IH vs. NC-PH	DC-PH vs. NC-PH		
Body weight, kg								
Birth (d 0)	37.2	37.1	37.7	0.66	0.98	0.74	<0.01	0.01
d 30	45.8	46.9	47.0	1.19	0.77	0.98		
d 60	63.7	65.8	66.2	1.20	0.48	0.91		
d 80	81.6	83.8	92.8	1.20	0.73	0.01		
ADG, kg								
d 0–30	0.278	0.314	0.327	0.04	0.57	0.90	<0.01	0.09
d 30–60	0.596	0.628	0.638	0.05	0.29	0.93		
d 60–80	0.892	0.898	1.332	0.02	0.79	0.02		
d 0–80	0.551	0.578	0.695	0.01	0.67	0.31		
Milk intake, kg DM/d								
d 0–30	0.616	0.616	0.775	0.01	1.00	0.05	<0.01	<0.01
d 30–60	0.759	0.759	1.073	0.02	1.00	<0.01		
Solid feed intake, kg DM/d								
d 0–30	0.078	0.063	0.045	0.01	0.78	0.71	<0.01	0.78
d 30–60	0.407	0.456	0.383	0.02	0.38	0.14		
Total intake, kg DM/d								
d 0–30	0.694	0.679	0.820	0.02	0.86	0.06	<0.01	0.05
d 30–60	1.166	1.215	1.460	0.03	0.57	<0.01		
Feed efficiency ^1^								
d 0–30	0.405	0.457	0.413	0.02	0.52	0.55	0.07	0.94
d 30–60	0.491	0.529	0.445	0.03	0.65	0.26		

* NC-IH = no dam contact, individually housed; NC-PH = no dam contact, pair-housed; DC-PH = dam contact (5 h/d), pair-housed. Sample sizes represent the number of calves originally allocated to each treatment group. Three calves died during the trial (two in DC-PH and one in NC-IH); data collected before mortality were retained in the analyses, whereas observations after death were treated as missing in the repeated-measures mixed model. ^1^ Calculated as kg weight gain/kg total DMI.

**Table 6:** Effect of dam contact and social companion on calf skeletal measures.

Items	Treatment Groups *			SEM	*p*-Value			
					Treatment		Time	Time × Treatment
	NC-IH (n = 8)	NC-PH (n = 8)	DC-PH (n = 14)		NC-IH vs. NC-PH	DC-PH vs. NC-PH		
Body length, cm								
Birth (d 0)	53.0	53.9	56.2	0.55	0.63	0.15	<0.01	<0.01
d 60	64.9	63.6	65.0	0.70	0.48	0.42		
d 80	69.3	69.3	72.7	0.71	0.99	0.05		
Hip width, cm								
Birth (d 0)	12.0	11.9	12.7	0.55	0.79	0.05	<0.01	0.99
d 60	15.9	16.3	16.5	0.21	0.51	0.71		
d 80	17.5	17.8	18.0	0.25	0.60	0.71		
Hip height, cm								
Birth (d 0)	76.9	76.3	78.6	0.55	0.74	0.18	<0.01	0.56
d 60	86.9	86.5	86.3	0.70	0.83	0.94		
d 80	91.0	90.6	91.3	0.71	0.82	0.67		
Withers height, cm								
Birth (d 0)	49.0	49.0	49.6	0.56	0.99	0.68	<0.01	0.02
d 60	54.7	55.5	54.9	0.64	0.64	0.70		
d 80	59.5	59.1	62.1	0.65	0.85	0.08		
Body barrel, cm								
Birth (d 0)	79.6	78.4	81.3	0.55	0.47	0.06	<0.01	0.65
d 60	99.7	101.4	99.9	1.25	0.62	0.64		
d 80	111.9	113.2	113.8	1.26	0.68	0.85		
Heart girth, cm								
Birth (d 0)	76.6	75.3	77.6	0.55	0.30	0.05	<0.01	0.03
d 60	95.0	95.9	91.9	0.95	0.73	0.10		
d 80	101.8	103.1	102.0	0.94	0.62	0.64		
Chest width, cm								
Birth (d 0)	8.62	8.63	9.43	0.55	0.99	0.20	<0.01	0.13
d 60	12.6	12.3	12.8	0.21	0.55	0.40		
d 80	13.6	13.4	14.3	0.21	0.71	0.09		

* NC-IH = no dam contact, individually housed; NC-PH = no dam contact, pair-housed; DC-PH = dam contact (5 h/d), pair-housed. Sample sizes represent the number of calves originally allocated to each treatment group. Three calves died during the trial (two in DC-PH and one in NC-IH); data collected before mortality were retained in the analyses, whereas observations after death were treated as missing in the repeated-measures mixed model.

**Table 7:** Effect of dam contact and social companion on selected blood metabolites and the number of protozoa in calf rumen.

Blood Metabolites	Treatment Groups *			SEM	*p*-Value			
					Treatment		Time	Time × Treatment
	NC-IH (n = 8)	NC-PH (n = 8)	DC-PH (n = 14)		NC-IH vs. NC-PH	DC-PH vs. NC-PH		
Glucose, mg/dL								
Pre-weaning, d 54	139.1	137.5	148.8	1.32	0.70	0.05	<0.01	0.66
Post-weaning, d 61	103.3	99.3	107.6	1.40	0.35	0.36		
βHBA, mmol/L								
Pre-weaning, d 54	0.090	0.093	0.058	0.01	0.89	0.06	<0.01	<0.01
Post-weaning, d 61	0.111	0.134	0.165	0.02	0.25	0.09		
Cortisol, µg/dL								
Pre-weaning, d 54	2.20	2.28	1.73	0.07	0.75	0.02	0.24	0.05
Post-weaning, d 61	2.12	1.80	2.05	0.08	0.20	0.26		
Protozoa numbers (×10^5^/mL)								
d 35	1.67	1.76	2.44	0.11	0.73	0.01	<0.01	0.04
d 70	2.34	2.93	4.03	0.11	0.04	<0.01		

* NC-IH = no dam contact, individually housed; NC-PH = no dam contact, pair-housed; DC-PH = dam contact (5 h/d), pair-housed. Sample sizes represent the number of calves originally allocated to each treatment group. Three calves died during the trial (two in DC-PH and one in NC-IH); data collected before mortality were retained in the analyses, whereas observations after death were treated as missing in the repeated-measures mixed model.

**Table 8:** Effect of dam contact and social companion on nutrient digestibility in calves.

Digestibility (%)	Treatment Groups *			SEM	*p*-Value	
	NC-IH (n = 7)	NC-PH (n = 8)	DC-PH (n = 12)		NC-IH vs. NC-PH	DC-PH vs. NC-PH
DM	81.4	87.7	88.1	1.40	0.03	0.84
NDF	46.5	60.3	68.4	4.07	0.13	0.46

* NC-IH = no dam contact, individually housed; NC-PH = no dam contact, pair-housed; DC-PH = dam contact (5 h/d), pair-housed. Digestibility measurements were obtained only from calves that remained alive until days 78–80. Therefore, sample sizes reflect the number of surviving calves.

## Data Availability

The original contributions presented in this study are included in the article. Further inquiries can be directed to the corresponding author.
